# Surgical versus conservative management of fifth metatarsal fractures in adults: A protocol of retrospective study

**DOI:** 10.1097/MD.0000000000022800

**Published:** 2020-10-16

**Authors:** Rongfu Qi, Bangnan Li, Tong Xie, Haijian Yin

**Affiliations:** Department of Orthopedics, Nanjing Tongren Hospital, School of Medicine, Southeast University, Jiangsu 211102, China.

**Keywords:** conservative treatment, fifth metatarsal fractures, protocol, retrospective, surgical management

## Abstract

**Background:**

At present, the treatment of base fractures of the fifth metatarsal, especially the area I fractures, is still a controversial topic. The objective of our work was to assess the radiological and clinical outcomes of displaced avulsion fractures of the fifth metatarsal base after treated with conservative treatment or intramedullary screw.

**Methods:**

All of the fifth metatarsal fractures patients underwent surgery by the senior authors in our hospital from January 2017 to December 2019 were reviewed. Institutional Review Board in the Subsidiary Hospital of Guizhou Medical University approved this study. Patients with the following conditions can be included:

(1) the displaced avulsion fracture of fifth metatarsal base;
(2) aged 18 to 40 years;
(3) a completed medical history;
(4) the patients were followed up for at least 6 months.

Patients with the following conditions will be excluded: follow-up less than 6 months; open fracture; pathological fracture; osteoporotic fracture; patients have the history of ankle or foot surgery. Radiographs were taken at 1, 3, 6 and 12 months in outpatient follow-up. The following outcomes were assessed in the follow-up: functional outcomes, the score of patient satisfaction, as well as the motion range, and complications. All the statistical analyses were implemented via applying the software of SPSS Version 12 (SPSS Inc, Chicago, IL).

**Results:**

We hypothesized that there was no remarkable difference between two groups in the outcomes after operation.

**Trial registration:**

This study protocol was registered in Research Registry (researchregistry6024).

## 1 Introduction

The fifth metatarsal fractures is one of the most familiar metatarsal fractures, with 6.7 fractures reported for every 10,000 people.^[1,2]^ The fracture of the proximal fifth metatarsal is not only owing to the direct injuries in this area, but also to the inverted injuries with plantar flexion. These fractures can lead to serious incidence rate, especially in athletes, the time of competition is obviously shortened, and refracture, sometimes it cannot resume sport.^[3–5]^

Generally speaking, the treatment of the fracture of the base of the fifth metatarsal is performed by non-surgical or surgical measures. Surgical procedures usually involve the bicortical screw or intramedullary internal fixation approaches.^[6–8]^ However, the non-surgical approach is to use the immobilization cast to promote the weight-bearing passive healing. At present, the treatment of base fractures of the fifth metatarsal, especially the area I fractures, is still a controversial topic.^[9–13]^ Some investigations have indicated that the conservative treatment may not yield the best long-term effects for athletic patients with displaced avulsion fracture of base zone I of the fifth metatarsal, leading to the delayed healing and resumption of activity.^[14–16]^ Furthermore, some literatures suggest the application of non-surgical intervention to promote rehabilitation. Studies have indicated that the application of non-surgical interventions can avoid surgery-related discomfort and the complications, which is a cost-effective method.^[17–20]^

The objective of our work was to assess the radiological and clinical outcomes of displaced avulsion fractures of the fifth metatarsal base after treated with conservative treatment or intramedullary screw. We hypothesized that there was no remarkable difference between two groups in the outcomes after operation.

## 2 Materials and methods

### 2.1 Patients

Patients with the following conditions can be included:

(1) the displaced avulsion fracture of fifth metatarsal base;
(2) aged 18 to 40 years;
(3) a completed medical history;
(4) the patients were followed up for at least 6 months.

Patients with the following conditions will be excluded: follow-up less than 6 months; open fracture; pathological fracture; osteoporotic fracture; patients have the history of ankle or foot surgery.

### 2.2 Study design

All of the fifth metatarsal fractures patients underwent surgery by the senior authors in our hospital from January 2017 to December 2019 were reviewed. Institutional Review Board in the Subsidiary Hospital of Guizhou Medical University approved this study (20201742). This study was registered in the research registry (researchregistry6024). This work was implemented and then reported on the basis of the requirement of strengthening the reporting of observational studies in epidemiology checklist (Table 1).

**Table 1:**
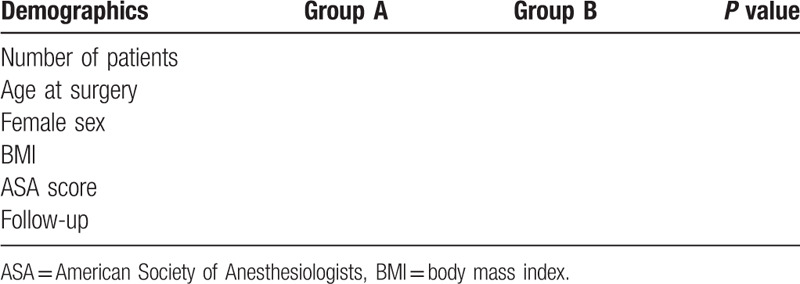
Patient baseline demographics.

### 2.3 Surgery and conservative technique

Each patient received a standard general anesthesia regimen and was placed on the radiologic operating table with supine position. This surgery was assisted through utilizin the tourniquet. And an approximately 3 cm longitudinal skin incision was made at the distal base of fifth metatarsal bone. After determining the lateral zone of aponeurosis and peroneus brevis, the periosteum was raised slightly to expose fracture. The reduction forceps are conducted by utilizing the fracture reduction. The screw drill was inserted through fracture site to determine the length of screw and the intramedullary purchase. The cannulated screw guide pin of 3 mm was inserted between the peroneus brevis tendon and the plantar aponeurosis lateral band.

In the conservative group, the patients were immobilized in the neutral position through using the plaster. The short leg plaster was utilized for 2 weeks, afterwards, before the weight bearing, the tubular plaster was applied for the next two weeks to four weeks. During immobilization, the hip joints and knee should be encouraged to exercise moderately to avoid lower extremity musculoskeletal atrophy and venous thrombosis. Moderate movements of the knee and hip were encouraged to avoid venous thrombosis and musculoskeletal atrophy of lower extremity during immobilization.

### 2.4 Postoperative rehabilitation

The passive and active flexion and extension of distinct ankle joints were carried out on the second day after operation. The patient was weight-free in the first 2 weeks and the incision was allowed to heal. As long as the sutures are removed at 2 weeks, the patient can bear the weight that the fracture boot can bear. At the same time, they can use swimming pool and land bike to gradually increase the resistance training for four weeks, and allow the complete weight-bearing at six weeks. All patients were asymptomatic in clinical and X-ray showed that after healing, they could resume complete activity.

### 2.5 Outcomes

Radiographs were taken at 1, 3, 6, and 12 months in outpatient follow-up. The examination of radiographs was conducted for the displacement of plate, implant failure, or signs of fracture healing. The following outcomes were assessed in the follow-up: functional outcomes, the score of patient satisfaction, as well as the motion range, and complications. The functional outcomes included Visual Analog Scale (VAS), Foot Function Index (FFI), as well as the American Orthopedic Foot and Ankle Score (AOFAS). Postoperative complications included infection, nonunion or delayed union, stiffness, refracture, and internal fixation loosening.

AOFAS is composed of 4 scoring systems, each of which containing two parts, in which the first part is subjective and it should be addressed via patient; the second part is objective and needs the clinical examination. In accordance with the affected anatomical area, the most relevant score should be given. This 4 scoring system included an examination of the alignment of anatomical joint. However, in these 4 systems, function item is divided into different and a variety of subitems. The total score is 100. The higher the obtained score, the better the patient's condition.

The FFI is composed of 23 items and is divided into 3 subscales: movement restriction, disability, and pain. The answers to each question ranges from 0 point (difficulty or no pain) to 10 point (so difficult help is needed or the most pain imaginable). The intensity of pain is assessed with the 100-mm horizontal VAS, where 0 mm was no pain and 100 mm was the extreme pain, and the level was compare between the groups.

### 2.6 Statistical analysis

The Student's unpaired *t* test and Chi-squared test were respectively applied to compare the qualitative outcomes and qualitative variables between the 2 groups. When the value of *P* was less than .05, it can be considered as significant in statistical. Power analysis was also carried out to determine if no remarkable differences were observed due to the small sample size. All the statistical analyses were implemented via applying the software of SPSS Version 12 (SPSS Inc, Chicago, IL).

## 3 Discussion

Owing to the special vascular system at the fifth metatarsal base, the fracture management is a challenging approach for orthopedics. It has been indicated in the literature that watershed-region at the fifth metatarsal base greatly reduces the success rate of fracture healing, particularly in the absence of surgical intervention. Therefore, in the past few decades, the application of surgical intervention in the treatment of fifth metatarsal base fractures has increased significantly. The application of invasive interventions is mainly welcome owing to they offer the rigid fixation of fracture surface while maintaining a retrograde blood supply to areas with insufficient blood vessels. The objective of our work was to assess the radiological and clinical outcomes of displaced avulsion fractures of the fifth metatarsal base after treated with conservative treatment or intramedullary screw. We hypothesized that there was no remarkable difference between two groups in the outcomes after operation.

## Author contributions

**Conceptualization:** Rongfu Qi, Bangnan Li

**Data curation:** Rongfu Qi, Bangnan Li

**Formal analysis:** Tong Xie

**Funding acquisition:** Haijian Yin

**Investigation:** Rongfu Qi

**Methodology:** Rongfu Qi, Tong Xie

**Resources:** Haijian Yin

**Software:** Bangnan Li, Tong Xie

**Supervision:** Bangnan Li, Haijian Yin

**Validation:** Tong Xie

**Visualization:** Bangnan Li

**Writing – original draft:** Rongfu Qi

**Writing – review & editing:** Haijian Yin

## References

[1] Wu GB Li B Yang YF Comparative study of surgical and conservative treatments for fifth metatarsal base avulsion fractures (type I) in young adults or athletes. J Orthop Surg (Hong Kong) 2018;26:2309499017747128. 29228848 10.1177/2309499017747128

[2] Cheung CN Lui TH Proximal fifth metatarsal fractures: anatomy, classification, treatment and complications. Arch Trauma Res 2016;5:e33298. 28144601 10.5812/atr.33298 PMC5251206

[3] Bernstein DT Mitchell RJ McCulloch PC Treatment of proximal fifth metatarsal fractures and refractures with plantar plating in elite athletes. Foot Ankle Int 2018;39:1410–5. 30079768 10.1177/1071100718791835

[4] Teoh KH Whitham R Wong JF The use of low-intensity pulsed ultrasound in treating delayed union of fifth metatarsal fractures. Foot (Edinb) 2018;35:52–5. 29793139 10.1016/j.foot.2018.01.004

[5] Thompson P Patel V Fallat LM Surgical management of fifth metatarsal diaphyseal fractures: a retrospective outcomes study. J Foot Ankle Surg 2017;56:463–7. 28476385 10.1053/j.jfas.2017.01.009

[6] Brogan K Bellringer S Akehurst H Virtual fracture clinic management of fifth metatarsal, including Jones’, fractures is safe and cost-effective. Injury 2017;48:966–70. 28284470 10.1016/j.injury.2017.02.003

[7] Kizaki K Yamashita F Mori D Ankle structures of professional soccer (Football) players with proximal diaphyseal stress fractures of the fifth metatarsal. J Foot Ankle Surg 2019;58:489–91. 30765251 10.1053/j.jfas.2018.09.024

[8] Mallee WH Weel H van Dijk CN Surgical versus conservative treatment for high-risk stress fractures of the lower leg (anterior tibial cortex, navicular and fifth metatarsal base): a systematic review. Br J Sports Med 2015;49:370–6. 25138980 10.1136/bjsports-2013-093246

[9] Pituckanotai K Arirachakaran A Piyapittayanun P Comparative outcomes of cast and removable support in fracture fifth metatarsal bone: systematic review and meta-analysis. J Foot Ankle Surg 2018;57:982–6. 30149851 10.1053/j.jfas.2018.03.018

[10] Zwitser EW Breederveld RS Fractures of the fifth metatarsal; diagnosis and treatment. Injury 2010;41:555–62. 19570536 10.1016/j.injury.2009.05.035

[11] Yates J Feeley I Sasikumar S Jones fracture of the fifth metatarsal: Is operative intervention justified? A systematic review of the literature and meta-analysis of results. Foot (Edinb) 2015;25:251–7. 26481787 10.1016/j.foot.2015.08.001

[12] Zhao J Yu B Xie M Surgical treatment of zone 1 fifth metatarsal base fractures using the locking compression plate distal ulna hook plate. J Am Podiatr Med Assoc 2017;107:369–74. 29077489 10.7547/15-208

[13] Polzer H Polzer S Mutschler W Acute fractures to the proximal fifth metatarsal bone: development of classification and treatment recommendations based on the current evidence. Injury 2012;43:1626–32. 22465516 10.1016/j.injury.2012.03.010

[14] Smith TO Clark A Hing CB Interventions for treating proximal fifth metatarsal fractures in adults: a meta-analysis of the current evidence-base. Foot Ankle Surg 2011;17:300–7. 22017907 10.1016/j.fas.2010.12.005

[15] Xie L Guo X Zhang SJ Locking compression plate distal ulna hook plate fixation versus intramedullary screw fixation for displaced avulsion fifth Metatarsal Base fractures: a comparative retrospective cohort study. BMC Musculoskelet Disord 2017;18:405. 28950848 10.1186/s12891-017-1766-z PMC5615762

[16] Kadar A Ankory R Karpf R Plate fixation of proximal fifth metatarsal fracture. J Am Podiatr Med Assoc 2015;105:389–94. 26429606 10.7547/14-035

[17] Seidenstricker CL Blahous EG Bouché RT Plate fixation with autogenous calcaneal dowel grafting proximal fourth and fifth metatarsal fractures: technique and case series. J Foot Ankle Surg 2017;56:975–81. 28606789 10.1053/j.jfas.2017.04.035

[18] Valkier C Fallat LM Jarski R Conservative versus surgical management of fifth metatarsal avulsion fractures. J Foot Ankle Surg 2020;59:988–92. 32684405 10.1053/j.jfas.2020.05.003

[19] Chuckpaiwong B Queen RM Easley ME Distinguishing Jones and proximal diaphyseal fractures of the fifth metatarsal. Clin Orthop Relat Res 2008;466:1966–70. 18363075 10.1007/s11999-008-0222-7 PMC2584274

[20] Mahajan V Chung HW Suh JS Fractures of the proximal fifth metatarsal: percutaneous bicortical fixation. Clin Orthop Surg 2011;3:140–6. 21629475 10.4055/cios.2011.3.2.140 PMC3095785

